# Impact of interdental brush shape on interpapillary cleaning efficacy – a clinical trial

**DOI:** 10.1038/s41598-020-64816-5

**Published:** 2020-05-13

**Authors:** Pune N. Paqué, Thomas Attin, Andreas Ender, Ahmad Al-Majid, Florian Wegehaupt, Beatrice Sener, Patrick R. Schmidlin

**Affiliations:** 1 Clinic of Conservative and Preventive Dentistry, Center of Dental Medicine, University of Zurich, Plattenstrasse 11, 8032 Zurich, Switzerland; 2 Dental department, King Faisal Hospital, Prince Muqrin St 1, 36361 Alhofuf, Saudi Arabia

**Keywords:** Tooth brushing, Preventive medicine

## Abstract

This study aimed to investigate whether interdental brush shape influences cleaning efficacy, by comparing a waist-shaped interdental brush (W-IDB) with a cylindrical IDB (C-IDB); both provided with the same bristle texture. Cleaning efficacy of differently shaped IDBs was measured in proximal surfaces of teeth in a split-mouth cross-over design. Twenty-eight patients abolished oral hygiene for 4 d. Line angle plaque area was scanned with an intraoral camera after use of disclosing dye in baseline and after IDB application and analyzed planimetrically. Additionally, bacterial load in the IDBs was analyzed after usage by colony forming units (cfu). A Wilcoxon signed-rank test with continuity correction was used to compare the results of the waist-shaped and the cylindrically-shaped IDBs. The waist-shaped IDBs cleaned significantly better than their cylindrically-shaped counterparts (area cleaned: 23.1% vs. 18.3%), when applied at same interdental spaces (p < 0.001). However, no significant differences were found in comparison of bacterial load on the IDBs (median cfu counts: 2.3E9 vs. 2.7E9, p = 0.93). Irrespective of bristle texture or size, IDB shape have impact on cleaning efficacy. Waist-shaped IDBs are more effective in cleaning of the line angle area than cylindrically-shaped IDBs.

## Introduction

Interdental cleaning has a pivotal role in establishing and maintaining optimal oral hygiene, mainly aiming to prevent gingivitis and periodontitis, and caries. Manual and electric toothbrushes are still incapable to adequately clean this region. *In-vitro* studies investigating interdental cleaning by hydrodynamic shear forces of powered toothbrushes (non-contact biofilm removal) indicate significant varieties between different types. Albeit results in interdental biofilm removal of side-to side toothbrushes (over their oscillating counterparts) seem promising, the interdental cleaning efficacy remains mainly supportive and seems still insufficient as such^1–6^. Therefore, additional tools for mechanical interdental cleaning, such as dental floss, tooth picks or interdental brushes (IDBs) have been developed and were investigated in several studies over the last decades: Evidence for a gingivitis-reducing effect was shown in randomized clinical trial by flossing and toothbrushing, to a minor extend also for plaque reduction^7^. However, no beneficial effect was found to reduce the risk of caries lesions by flossing^8–12^. The efficacy of wood sticks was analyzed in different clinical studies, also showing interdental gingivitis reduction; no reduction in plaque nor any evidence for caries prevention could be elaborated^13^. The only interdental cleaning device so far, which was able to reduce plaque accumulation, bleeding scores, and probing pocket depth effectively alike in clinical trials were IDBs^7^. Larsen *et al*. compared the effectiveness of conically-shaped and cylindrically-shaped interdental brushes in a randomized controlled clinical trial^14^. Not surprisingly, conical IDBs resulted in significantly higher plaque and bleeding scores especially at the lingual approximal sites due to the fact that brush size diameter was not effective anymore to reach and clean these aspects. In contrast, another study compared waist- and cylindrically-shaped IDBs^15^. Statistically significant differences in plaque reduction after IDB application were observed for both IDB types. However, especially the areas between the lingual line angles resulted in significantly lower plaque when the waist-shaped IDBs were used. Both studies indicated that an effect of IDB shape in cleaning efficacy can be expected. However, aside from IDB shape and size, only little is known about bristle texture, central wire, and filaments of applied IDBs. Noteworthy in this context, the latter study applied standardized IDB sizes for all subjects without taking the appropriate IDB sizes for each individual interdental space into account. While Larsen and co-workers^14^ adjusted IDBs individually, the quality of the user’s technique in interdental cleaning was not taken into account.

This study now aimed to investigate the impact of interdental brush shape under standardized conditions, comparing a waist-shaped interdental brush (W-IDB) with a cylindrical IDB (C-IDB) with the identical wire and bristle architecture, but mainly different shape. Cleaning efficacy in the line angle area was evaluated after adjusting each IDB size individually for each interdental space (individual best fit as shown in the study of Larsen and co-workers^14^). The actual cleaning procedure applying IDBs was, however, performed under standardized conditions by one trained dentist. The volume capacities of each IDB size were analyzed assessing the bacterial load of applied interdental brushes. The null hypothesis was that the different IDBs show no statistically significant different cleaning results when professionally used in a best fit-approach.

## Materials and Methods

The study was approved by the Ethics Committee Zurich (BASEC-no. 2016-00266) and registered in the Internet Portal of the German Clinical Trials Register (DRKS00009823, date of first registration: 05/08/2016) as well as in the Swiss National Clinical Trials Portal (SNTP000001645). Written informed consent was obtained from all participants included in this study.

### Study population

**Figure 1:**
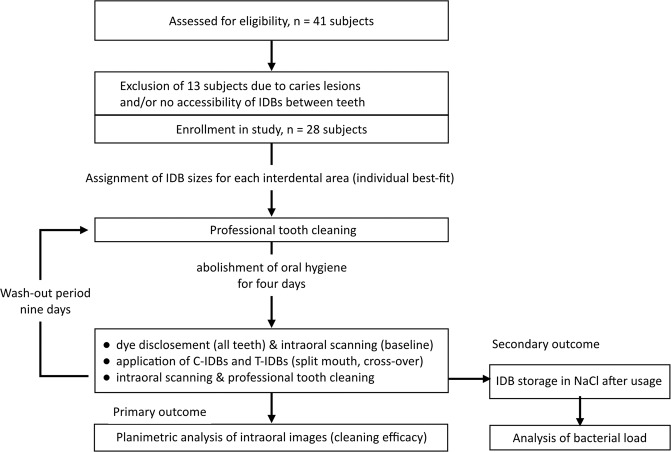
Flow chart with details on subject enrollment and intervention in this study.

Systemically healthy subjects with at least three adjacent posterior teeth on both maxillary quadrants. Only sites nearly free of approximal restorations (a maximum of two approximal restorations and/or two restorations at the buccal sites were eligible) were included in this study in order to avoid plaque retentive niches and surfaces, which might hamper the disclosing and cleaning, respectively. Further inclusion criteria were: Otherwise caries-free dentition, GI scores ≥ 1 and free accessibility of the interdental area with brushes in three approximal areas on both maxillary sites. Exclusion criteria were: Participation in any other clinical trial within the last three months prior to study enrollment, alcohol, drug, or medicament abuse, heavy smoking (> 10 cigarettes a day) and willingness to comply with the study requirements. Subjects taking antibiotics during the study were not allowed to participate in order to standardize the systemically healthy state and minimize bias in the bacterial analysis of the IDBs. None of the volunteers, however reported any antibiotic intake in the last three months before the study. Sample size was set to at least 16 subjects in order to ensure comparability other studies^14,15^, which served as reference. The trial was designed to end in December 2016, due to local logistics at the clinic and the dense appointments with respective wash-out phases. Subjects were recruited from July to November 2016 at the Center for Dental Medicine, Zurich, Switzerland by flyers at local university facilities and the website. Forty-one healthy subjects were interested to participate and were initially screened for inclusion/exclusion criteria. Twenty-eight healthy volunteers aged 18–59 (mean: 28.9 years; stratified for gender, 14 males) were enrolled in the trial, starting with interventions from July until December 2016 (Fig. 1). All twenty-eight subjects appeared for all interventions and follow-up tooth cleaning.

### Study design

The study was an interventional controlled, stratified, split-mouth cross-over study at one center (Center for Dental Medicine, University of Zurich, Switzerland). All subjects were asked to attend five appointments. At the first visit, all subjects were informed about the study details and screened for potential in- and exclusion criteria. The fitting size of the interdental brushes (waist-shaped or cylindrical) was determined independently for each approximal region (i.e. six per subject). This was performed by one trained dentist. At the second and fourth visit, subjects received a professional tooth cleaning. Then, volunteers were asked to abolish oral hygiene for four days. At the third and fifth visit, plaque accumulation was disclosed with a dye (paro® plak, Esro AG, Kilchberg, Switzerland) and the defined proximal regions were recorded as baseline with an intraoral scanning device (3Shape Trios, TRC; 3Shape, Copenhagen, Denmark). One maxillary side was then allocated to be interproximally cleaned with waist-shaped interdental brushes, whereas cylindrically-shaped interdental brushes were applied on the other side (split-mouth). At the fifth visit, sides were switched and the remaining brush type was used (cross-over design). This enabled the testing of both groups (C- and W-IDBs) in each subject simultaneously. Furthermore, the cross-over design minimized possible bias by maxillary side differences, since all interdental spaces were cleaned by both groups at the end. Approximal cleaning with C- and W-IDBs was performed again by the same trained dentist, who already determined the IDB size. IDBs were applied five times through the interdental spaces. After application, the brush heads were stored in 0.9% sodium chloride (NaCl) filled tubes. After cleaning, interdental spaces were scanned again with the intraoral camera after IDB application. A professional tooth cleaning was performed at the end of the third and fifth visit. All professional tooth cleaning procedures (second, third, fourth, and fifth visit) were performed by a dental hygienist or trained dentists and took up to 30 min per subject. In between the visits, a wash-out period of nine days was interposed (Fig. 1). The final analysis of cleaning efficacy and colony forming units was performed under blinded conditions by the study personnel.

### Interdental brushes

**Figure 2:**
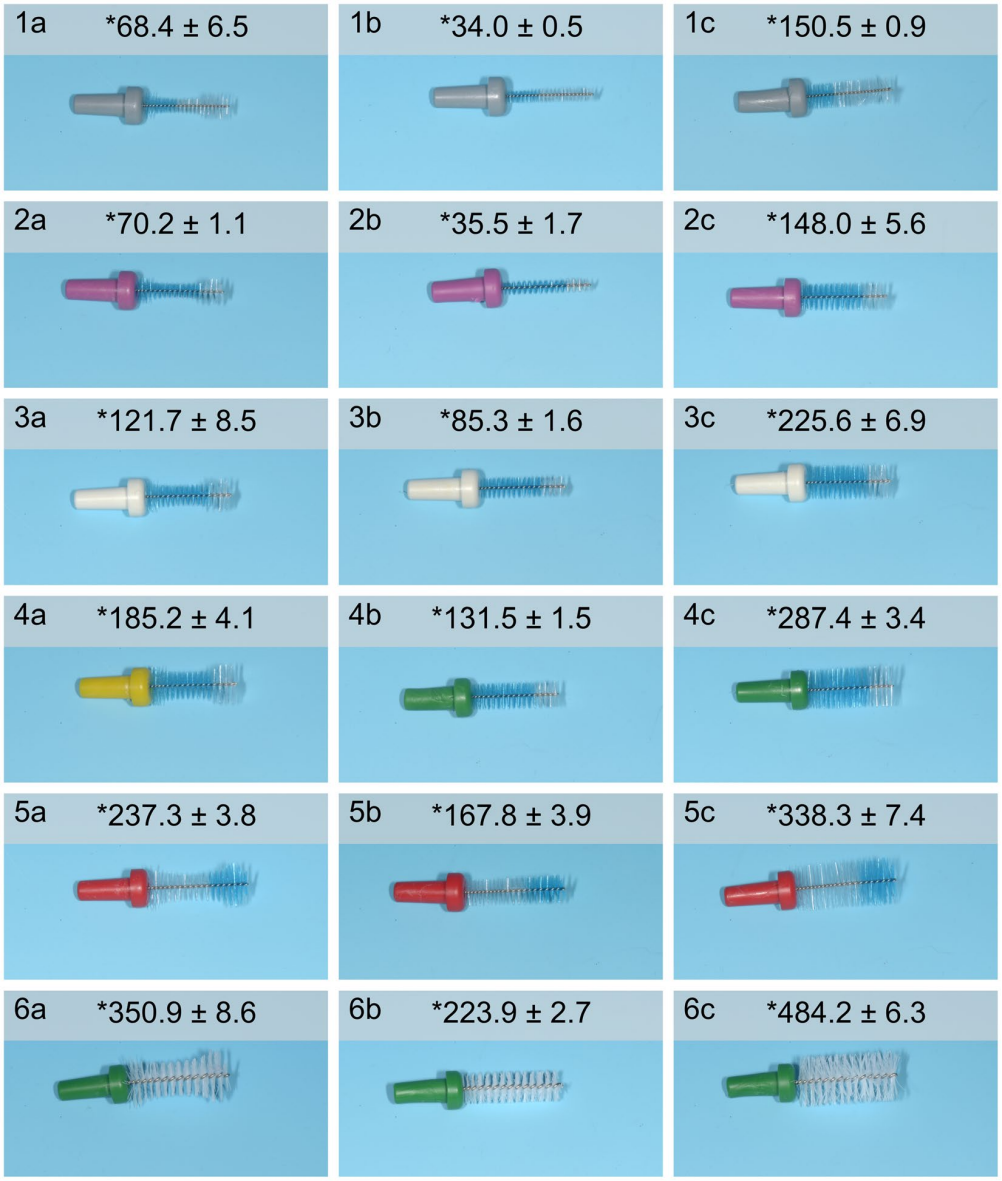
Representative images of waist-shaped (W-IDB, 1a – 6a) and cylindrically-shaped interdental brushes (C-IDB). C-IDBs were manufactured as counterparts of W-IDBs with the same bristle texture with a minimum W-IDB diameter (1b – 6b) and maximum W-IDB diameter (1c – 6c). Average bristle volume capacity ± deviation of each IDB are expressed in mg and marked with asterisks.

Waist-shaped interdental brushes (Circum®, Top Caredent, Zürich, Switzerland) were compared with custom-made cylindrically-shaped interdental brushes with identical bristle texture. Waist-shaped brushes of size 1–6, and 12 corresponding cylindrically-shaped brushes were used in this study (Fig. 2). Therefore, the cylindrically-shaped IDBs were manufactured as counterparts of the waist-shaped IDBs with the bristle lengths according to the minimum and the maximum of the waist-shaped IDB bristle diameter. IDB sizes for both groups were determined for each interdental space and subject individually. As pilot experiment, an *in vitro* evaluation of cleaning efficacy between both groups was performed prior to the clinical study^16^. Due to different IDB shapes used (waist-shaped and cylindrical IDBs), a volume analysis of the bristle capacity was performed to allow a comparison of the potential fluid uptake under standardized conditions between the different applied IDB shapes. For this purpose, all IDB sizes of both groups were analyzed by a weight assay. In short, this means that the volumes of all IDB shapes and sizes applied in this study were calculated by wetting the IDBs with an adhering solution. The corresponding weight prior to and after artificial wetting (5 repetitions) was used to calculate the respective volume capacities as a surrogate parameter for saliva and plaque uptake, when applying the brushes. The applied solution was a hydroxyethyl-cellulose solution (1% in Aqua dest. 20.5 °C). Volume capacities [mg] were calculated considering density and used as surrogate quantification method for maximal plaque admission by IDBs. The calculated volumes are included in Figs. 5 and 7 to illustrate cleaning efficacies and bacterial load correlating to the IDB sizes and shapes applied.

**Figure 3:**
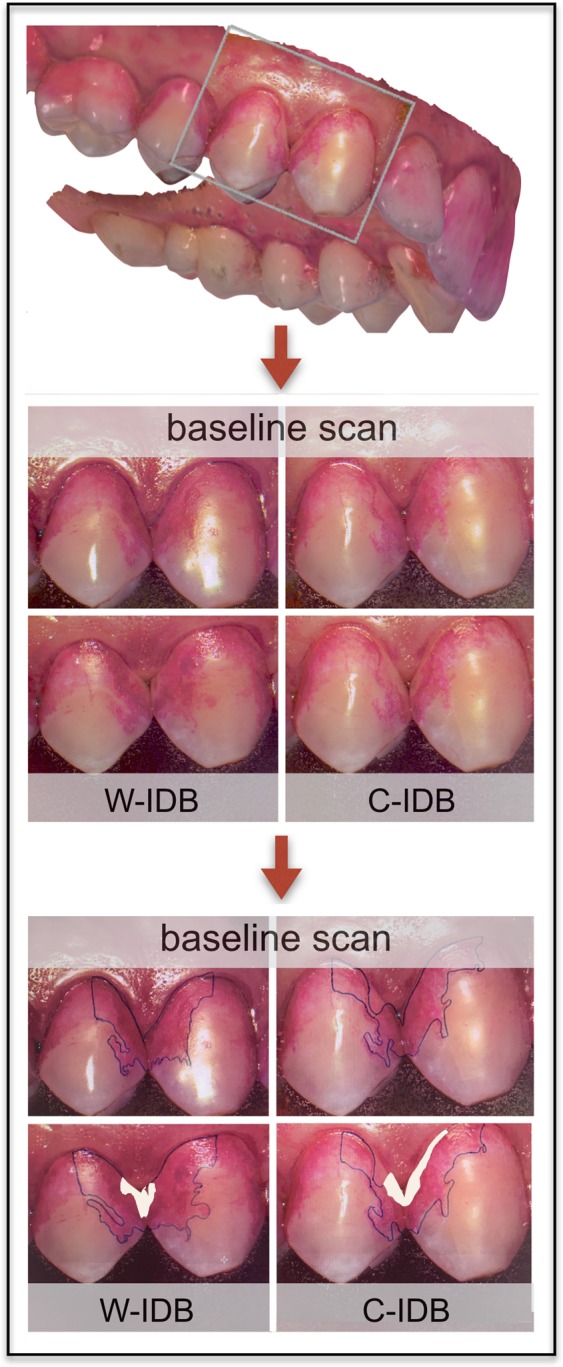
Cleaning efficacy procedure for one representative interdental space using intraoral images taken before (baseline) and after W- and C-IDB application (W-IDB = waist-shaped, C-IDB = cylindrically-shaped interdental brush). Images were analyzed planimetrically by marking colored plaque accumulation in the line angle area on each image, and depicting the cleaned region (marked in black) on post-brushed images.

**Figure 4:**
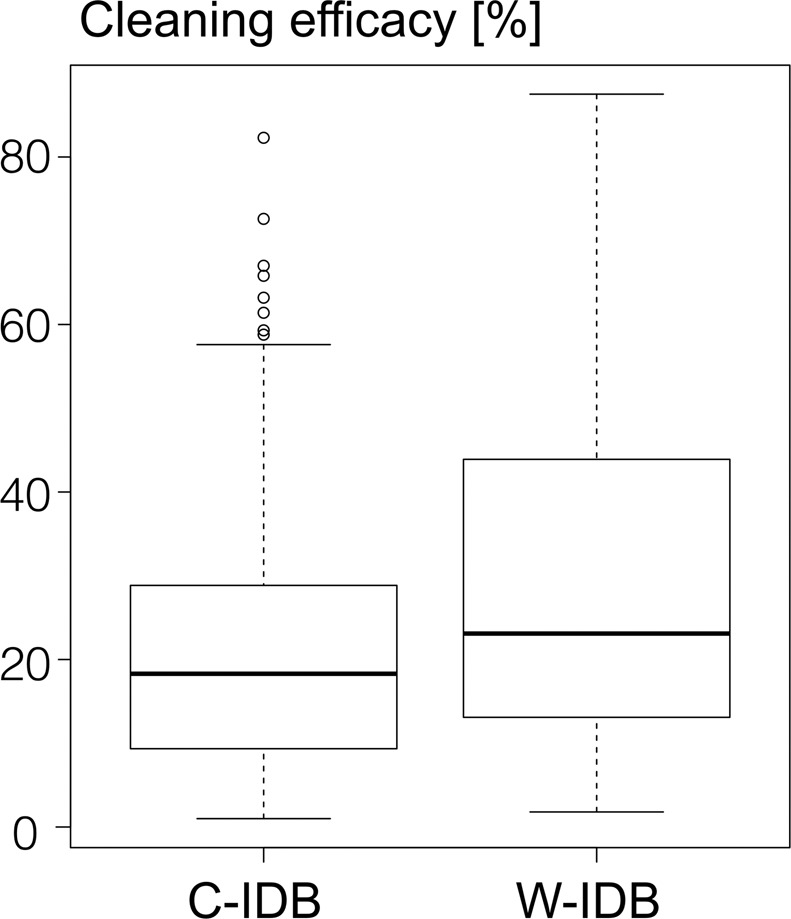
Boxplot of cleaning efficacy [%] of waist-shaped (W-IDB) and cylindrically-shaped (C-IDB) interdental brushes. Boxplots showed median percentages, 25^th^ and 75^th^ quartiles, standard deviation and outliers for cleaned areas (percentages) (in an interdental-space-wise comparison).

**Figure 5:**
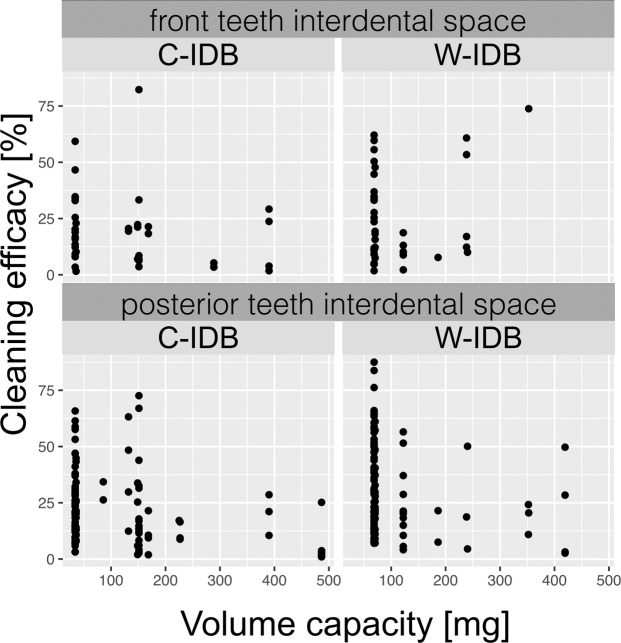
Dotplots of cleaning efficacy data [%] (waist-shaped (W-IDB) and cylindrically-shaped (C-IDB) interdental brushes) at different volume capacities [mg] of all applied interdental brushes. Data is separately analyzed for interdental spaces in anterior (intercanine region) and in posterior teeth (premolars, molars).

**Figure 6:**
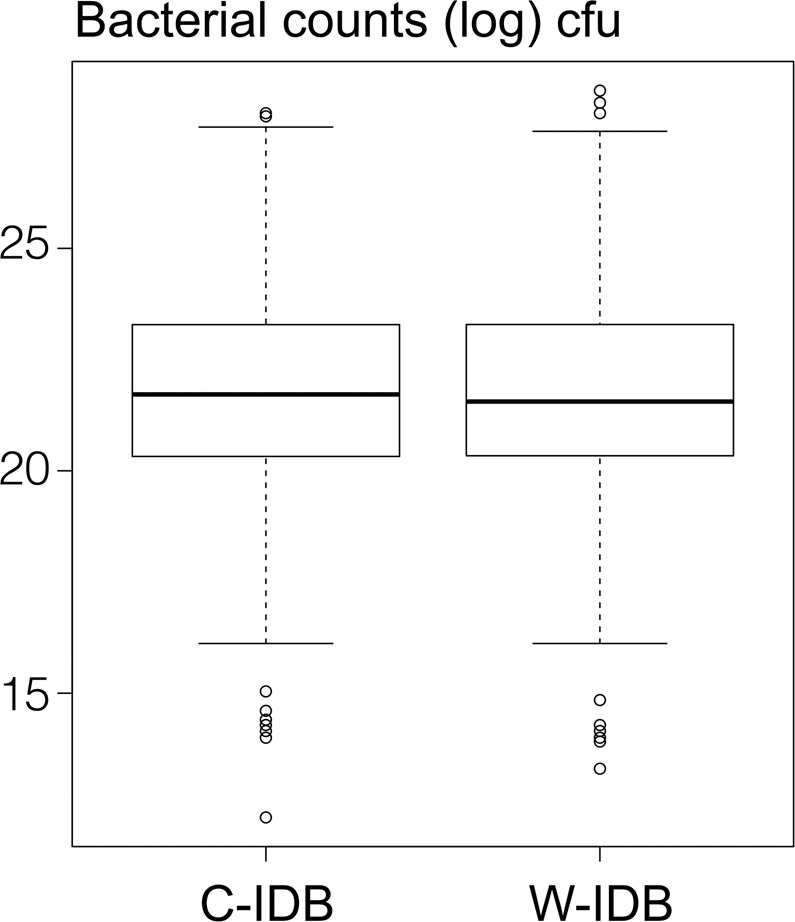
Boxplot of bacterial counts (log cfu) for waist-shaped (W-IDB) and cylindrically-shaped (C-IDB) interdental brushes. Boxplots give median (log) cfu, 25^th^ and 75^th^ quartiles, and standard deviation and outliers.

**Figure 7:**
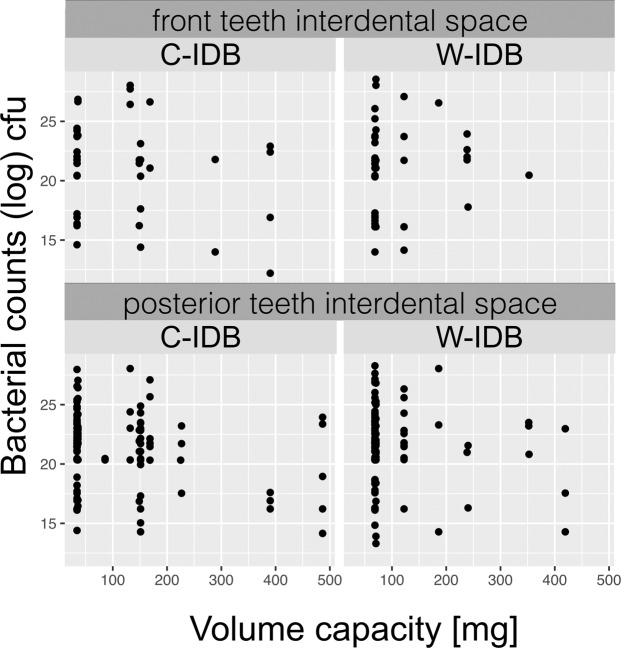
Dotplots of cfu data [log(cfu)] (waist-shaped (W-IDB) and cylindrically-shaped (C-IDB) interdental brushes) at volume capacities [mg] of all applied interdental brushes. Data is separately analyzed for interdental spaces in anterior (intercanine region) and in posterior teeth (premolars, molars).

### Cleaning efficacy

Cleaning efficacy was analyzed as primary outcome to investigate potential differences in applied brush shapes. Intraoral images of each proximal region were taken with an intraoral scanning device (3Shape Trios) at baseline and after IDB application. Each interdental space was scanned at least twice. Images before and after cleaning were planimetrically analyzed (Digitizer-Planimeter, Textronix Inc. Beavertn, Oregon, USA) and expressed as absolute value in mm^2^ and as percentage of the entire line angle area (Fig. 3). Therefore, the colored plaque accumulation in the line angle area was marked on each single image (baseline and post-brushing). A validated custom-made software with a grey-scale threshold^16,17^ was applied to calculate the plaque-covered surfaces to mm^2^ (calibrated by a canine width of 5 mm). The baseline surfaces were set to 100% and the surfaces of the post-brushing images were calculated accordingly. Altogether, 336 interdental spaces were analyzed. The images were coded and anonymized for this evaluation by a combination of the interdental space analyzed, the initials of the pre- and surname, and the number of subject enrollments in the study, as well as the date of the intervention. Decoding was possible by a key document (accessible for the trained dentist who performed the interventions), containing information of the examination of each single subject. After analysis and statistics, the values were decoded and results assigned to brush shape.

### Colony forming units

Bacterial load was assessed as secondary outcome in this study. IDBs of both types were stored at 5 °C in 1 ml of 0.9% sterile saline solution after usage as mentioned above. Colony forming units (cfu) were determined on the same day: In short, vials with IDBs were vortexed for 1 min to homogenize bacterial aggregates, and disperse bacteria, which adhered on the bristles of the interdental brushes after the cleaning process. The suspension was then processed in a dilution series of 10^−4^, 10^−6^, and 10^−8^ with 0.9% sterile saline solution. Then, 50 µl of each dilution was pipetted on Columbia sheep’s blood agar (CSBA) plates (bioMérieux, Marcy l’Etoile, France). CSBA plates were incubated anaerobically in jars with gas-paks (GENbag anaer, bioMerieux) at 37 °C for two days. Colonies were counted after two days and expressed as CFU/ml. Again, samples were coded for the laboratory evaluation and the statistician deciphered the results after analysis to the brush shape used.

### Statistical analysis

Cleaning effects (% of cleaned area in the line angle region) and bacterial load (median cfu counts) after application of either waist-shaped or cylindrically-shaped IDBs were compared for each interdental space separately. The difference of cleaning efficacy per tooth and interdental space was calculated between waist-shaped and cylindrically-shaped IDBs. This difference was tested using a Wilcoxon signed-rank test with continuity correction. In addition, IDB sizes (presented as calculated volumes) of both groups were correlated with median results of bacterial volume and cleaning efficacy for anterior and posterior teeth separately. The significance level was set to α = 0.05. The weighted means of all applied IDBs were calculated for anterior and posterior teeth. All calculations were performed with the program R^18^. Interquartile ranges (IQR) were calculated and presented in graphs.

### Ethical approval

All procedures performed in this study involving human participants were in accordance with the ethical standards of the institutional and/or national research committee and with the 1964 Helsinki declaration and its later amendments or comparable ethical standards.

### Informed consent

Informed consent was obtained from all individual participants included in the study.

## Results

The outline of the study is presented in Fig. 1. Thirteen subjects did not fulfill the inclusion criteria; hence 28 subjects could be finally enrolled in this trial, of which all completed the study. Altogether, 40 anterior (between incisors and canines) and 128 posterior (canines to molars) interdental regions were assessed. The most frequently analyzed interdental spaces were located in posterior teeth, between canines and the first premolar (n = 106), the first and second premolar (n = 76), and the second premolar and first molar (n = 70). The interdental brush sizes used in this study were converted for all interdental spaces (anterior and posterior teeth) in volumes. The weighted means of the differently-shaped interdental brushes were 104.5 ± 110.5 mg for cylindrically-shaped and 101.5 ± 76.8 mg for waist-shaped IDBs, respectively. Higher IDB volumes were used for the cleaning of interdental spaces in anterior teeth (C-IDB: 123.6 ± 113.5 mg; W-IDB: 107.5 ± 69.3 mg) as compared to posterior teeth (C-IDB: 98.0 ± 108.5 mg; W-IDB: 99.2 ± 78.9 mg).

### Cleaning efficacy

Comparing the cleaned area (percentage of baseline) after application of the waist-shaped with the cylindrically-shaped IDB of each interdental space resulted in a significantly better (p < 0.001) cleaning efficacy of waist-shaped IDBs (Fig. 4): The cleaning performance (cleaned area, median values and IQR in brackets) of waist- and conically-shaped IDBs accounted for 23.1% (30.8%) and 18.3% (IQR 19.5%). The mean and standard deviation of the cleaning efficacies were 21 ± 16% for the C-IDBs and 29 ± 19% for the W-IDBs. Posthoc power was calculated as being 99%, thus indicating that the sample size was generous and could possibly be reduced in similar follow-up studies. The group-wise correlation of anterior and posterior teeth and the treated interdental spaces with the measured IDB volumes illustrates the cleaning pattern with respect to IDB sizes of both test groups (Fig. 5). The distribution of interdental brush sizes (volume capacity) of both groups is presented as dotplots for anterior and posterior teeth and in correlation to the cleaning efficacy in percentage (Fig. 5). W-IDBs resulted in higher cleaning percentages in both areas, irrespective of the volume capacity. IDBs with volumes >200 mg showed only reduced cleaning efficacy in conically-shaped IDBs with less than 30% cleaning in line angle areas, whereas waist-shaped IDBs showed partially more than 50% cleaned areas in anterior teeth. However, due to a low number of applied high volumes in anterior and posterior teeth (e.g. application of high IDB sizes in the study), a higher cleaning efficacy is predicated on smaller IDB sizes (up to 100 mg, corresponding to W-IDB size 1 and 2).

### Colony forming units

Culturing bacteria from the applied IDBs showed no statistical differences between test IDBs (p = 0.93) (Fig. 6). The median cfu counts of waist-shaped IDBs resulted in 2.3E9, IQR 1.2E10, and of cylindrically-shaped IDBs in 2.7E9, IQR 1.2E10. The mean and standard deviation of the bacterial load (cfu counts) were 6.2E10 ± 2.3E11 for the C-IDBs and 8.0E10 ± 3.0E11 for the W-IDBs. A correlation of bacterial load and IDB size (volume capacity) shows no distinct differentiation between waist-shaped and cylindrically-shaped IDBs nor between interdental spaces of anterior and posterior teeth (Fig. 7). The dotplots show graphically how many IDBs of which size (volume capacity) were used in anterior and posterior teeth (Fig. 7), and their bacterial load after cleaning (log cfu). A tendency was found in in both groups (C- and W-IDBs) to show a higher bacterial load with smaller IDB sizes (<200 mg volume capacity, corresponding to W-IDB sizes 1–4).

## Discussion

The impact of interdental brush shape was determined in this study in a split-mouth cross-over design. The results have shown that waist-shaped interdental brushes were more effective in cleaning the interdental line angle areas of healthy subject than cylindrically-shaped IDBs with the same bristle texture. The null hypothesis was therefore neglected.

Till to date, different studies compared the impact of IDB shape by comparing different brands of IDBs with varying filament arrangements and bristle architecture. Thus, studies were not capable to reach a definitive conclusion on the single comparative effect of IDB shape as such^14,15^. Analyzing the impact of IDB shapes without exerting the influence from different wires and fibers, required the individual manufacturing of identical IDBs with different shapes. For this study, commercially available waist-shaped IDBs were compared with their identical cylindrically-shaped counterparts, thus only differing in shape and size. In order to eliminate further bias, IDB sizes were adjusted for all IDBs separately in all interdental regions and a volume capacity test of applied IDBs resulted in a comparable distribution. It should be mentioned, that the actual cleaning with IDBs was performed by one trained dentist and not by the subjects themselves. This procedure ensured a standardized IDB application throughout the study. However, two critical aspects with this study approach should be highlighted: (1) the trained dentist, who performed the cleaning with the IDBs was not blinded during the study; (2) the manual skills of the individual subjects were not considered in the obtained results. The blinding of the examiner was simply not possible due to the handling of the actual cleaning with the interdental brush. The subjects, however, were blinded and more importantly in this study, the scans (for the planimetric analysis of the cleaning efficacy) and all samples (used IDBs for microbiological analysis) were coded. Therefore, the complete analysis was a blinded procedure. The lack of letting manual skills of the subjects influence the outcome of the cleaning was intended to standardize the procedure as much as possible.

For the analysis of the cleaning efficacy, colored plaque was marked to calculate the plaque-covered surfaces in mm^2^ by the custom-made software. A calibration for assessing the outcome by means of distinguishing the red-colored tooth area was not performed, since a clear differentiation between red and the tooth color was possible after 4 days of plaque accumulation. Nonetheless, follow-up studies could be improved by an a priori calibration.

IDB application is mainly indicated in open embrasure spaces, implants or dental prosthesis, and wide interdental spaces, which are more often found in an elderly population or in patients suffering from periodontitis^7,19^. However, all participating subjects enrolled in this study were in a good general state of health, without caries or gingival inflammation, with a mean age of 28.9 years.

Altogether, 28 subjects were enrolled in this study, which is more than in the study of Larsen and co-workers^14^, who analyzed 25–26 subjects per group and of Chonghcharoen *et al*.^15^, who included only eight subjects in total, however, thereby analyzing both jaws, the maxilla and the mandible. It was, however, a challenge to find suitable subjects, exhibiting interdental brush- accessibility in three proximate sites per maxilla side. Hence, most applied IDB sizes were rather small (volume capacity <200 mg, corresponding to IDB sizes 1–4). Highest differences between waist-shaped and cylindrically-shaped IDBs were also found for smaller IDBs due to availability. In further studies, it would be interesting to evaluate, whether differences in cleaning efficacy would further increase, if more IDBs with higher sizes could be included. Interestingly, higher IDB sizes were applied in this study for anterior teeth compared to posterior teeth. This may be anatomically explained by the higher contact area of posterior teeth in interdental regions, which may reduce or restrict the interdental space. In contrast, the contact area of anterior teeth is more linear and may allow bigger IDBs to pass through. Due to the disclosing dye, a high cleaning of IDBs was also observed on gingival regions below the line angle area. It is possible, that IDB bristles would reach deeper areas in periodontitis patients, leading to higher cleaning efficacies. However, it is not clear, if a planimetrical analysis as used in this study would be adequate for those measurements as well. For healthy subjects without gingival inflammation, however, the planimetric analysis seemed more precise than other plaque indices. This is due to the area-focused plaque analysis compared to plaque indices, where solely the presence or absence of plaque per tooth side are measured^14^. Larsen and co-workers^14^ analyzed the long-term application of different brands and let subjects use their IDBs themselves. In the study of Chongcharoen *et al*.^15^ however, the IDBs were applied only once by a trained dental surgery assistant. Hence, bleeding on probing indices were not appropriate in this analysis and the influence of the manual skills of the subjects were also not included in the study. This is also in line with the present study, where interdental brushes were only applied once.

A limitation in this study concerns the lack of randomization of the cross-over sequences. The W-IDBs were applied on all three interdental spaces of the right maxilla in the third visit and the C-IDBs in all interdental spaces of the left maxilla. In the fifth visit, the W-IDBs were used in the left maxilla, and C-IDBs in the right maxilla. A proper randomization by means of a maxilla-wise or even interdental region-dependent allocation was not performed. However, it is highly unlikely, that this may have introduced significant bias, because both IDB groups were applied to either side of the mouth of each patient. This within-subject design avoids most sources of bias and ensures high statistical power. In addition, in this study, different sizes of interdental brushes of each group were assigned to each interdental space individually, in contrast to the study of Chongcharoen *et al*.^15^ were only two different sizes were applied for all interdental spaces.

As second outcome, bacterial loads were also assessed. The method of cfu measurements on IDBs after application is new and was tested in preliminary settings. No significant differences were found in the results with respect to total cfu’s (colony forming units). Though, interestingly, slightly more bacteria were detected in smaller (<200 mg volume capacity) than in higher IDB sizes for both groups. A limiting factor of this study and evaluation method, it is worth to mention that not all bacteria can be cultured. A microscopic quantification of all bacteria after adequate fluorescence staining techniques would potentially be more precise^20^. However, this was far beyond the scope of this study and finally, both cleaning methods were evaluated with comparable methods, which may nevertheless allow for an adequate comparison. Unfortunately, there are no comparable studies using this technique as surrogate for interdental cleaning procedures and measurements involving the *in vivo* state of health (here teeth and/or gum condition). Additional research on this topic is of interest and studies are planned.

## Conclusion

Irrespective of bristle texture or size, IDB shape is found to influence cleaning efficacy in healthy subjects and under standardized conditions, when comparing waist-shaped with cylindrically-shaped interdental brushes. Waist-shaped IDBs were more effective in cleaning the line angle area than cylindrically-shaped IDBs.

## Data Availability

The datasets generated during and/or analyzed during the current study are available from the corresponding author on reasonable request.
